# Chromosome-level genome assembly of the cottony cushion scale *Icerya purchasi*

**DOI:** 10.1038/s41597-024-03502-x

**Published:** 2024-06-17

**Authors:** Jun Deng, Lin Zhang, Hui Zhang, Xubo Wang, Xiaolei Huang

**Affiliations:** 1 State Key Laboratory of Ecological Pest Control for Fujian and Taiwan Crops, College of Plant Protection, Fujian Agriculture and Forestry University, Fuzhou, 350002 China; 2 Key Laboratory of Forest Disaster Warning and Control in Yunnan Province, College of Biodiversity Conservation, Southwest Forestry University, Kunming, 650224 China; 3 Yunnan Academy of Biodiversity, Southwest Forestry University, Kunming, 650224 China

**Keywords:** Entomology, Genome

## Abstract

The cottony cushion scale, *Icerya purchasi*, a polyphagous pest, poses a significant threat to the global citrus industry. The hermaphroditic self-fertilization observed in *I. purchasi* is an exceptionally rare reproductive mode among insects. In this study, we successfully assembled a chromosome-level genome sequence for *I. purchasi* using PacBio long-reads and the Hi-C technique, resulting in a total size of 1,103.38 Mb and a contig N50 of 12.81 Mb. The genome comprises 14,046 predicted protein-coding genes, with 462,722,633 bp occurrence of repetitive sequences. BUSCO analysis revealed a completeness score of 93.20%. The genome sequence of *I. purchasi* serves as a crucial resource for comprehending the reproductive modes in insects, with particular emphasis on hermaphroditic self-fertilization.

## Background & Summary

The cottony cushion scale, *Icerya purchasi* Maskell (Monophlebidae: *Icerya*), is a destructive pest well-known for infecting citrus and lemon plants, with a wide distribution across approximately 150 countries in America, Asia, Europe, and Oceania^1^. Understanding the observed diversity in the reproduction modes across different taxa has become a key focus in evolutionary biology. Scale insects (Hemiptera: Sternorrhyncha: Coccoidea), comprising 8535 species, exhibit significant variation in their reproduction modes, including paternal genome elimination, haplodiploidy, parthenogenesis, diplodiploidy, and hermaphroditism^2,3^. *I. purchasi* displays a unique reproductive mode among insects, which is rare in arthropods^4,5^. Hermaphrodites with a female-like appearance produce sperm and undergo self-fertilization^6^. Male individuals are scarce in the wild and occasionally mate with hermaphrodites, highlighting the colonization advantages of a selfing organism and the periodic reintroduction of genetic variation^6^. To date, there are seven chromosome-level genomes belonging to scale insects available from GenBank, two of which have been published as research papers^7,8^. A high-quality reference genome of *I. purchasi* would be invaluable for comprehending the mechanism of hermaphroditic self-fertilization.

**Fig. 1:**
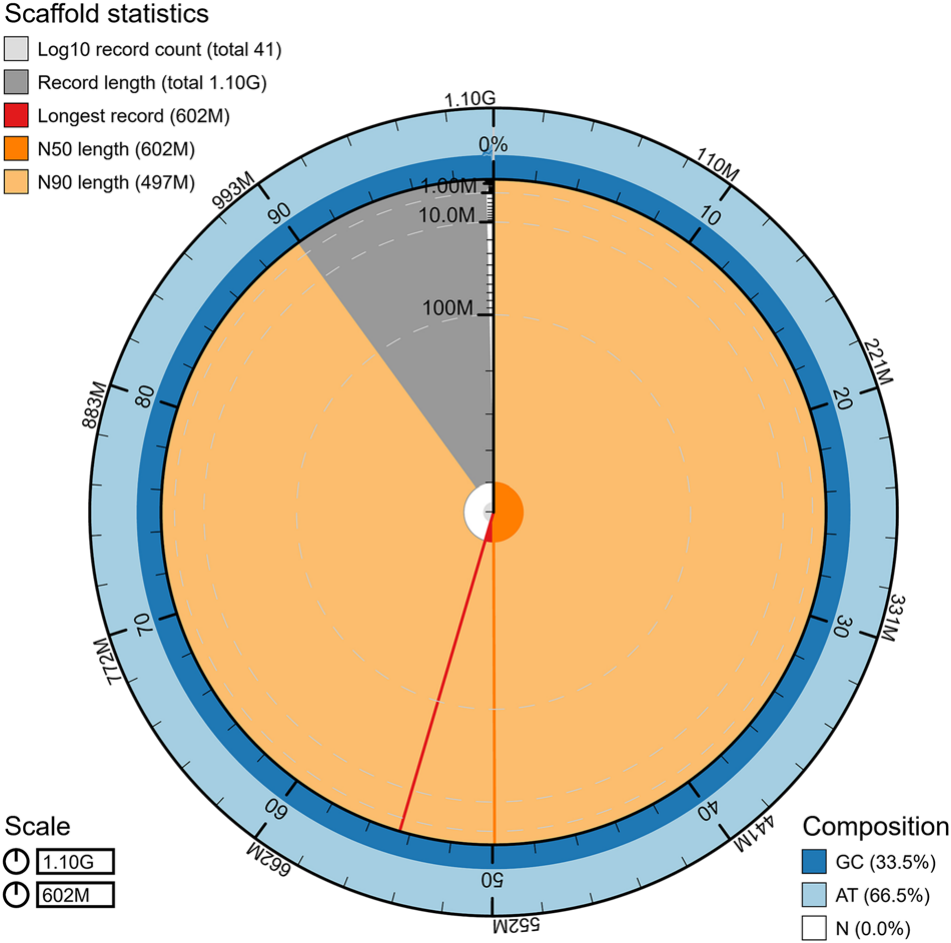
The snail plot shows metrics of the *I. purchasi* genome including the length of the longest contig, N50, N90, base composition.

**Fig. 2:**
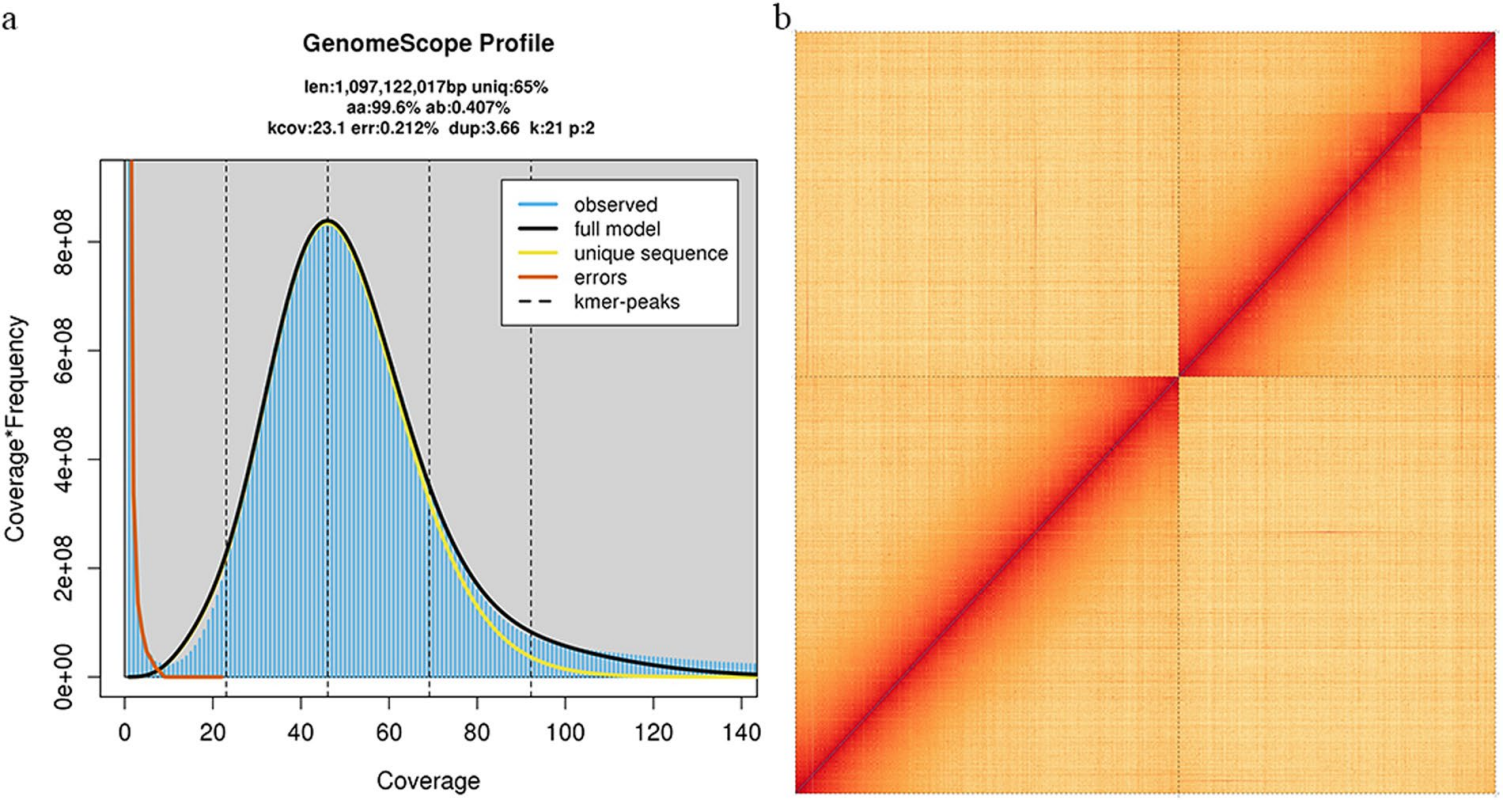
(**a**) Estimated genome size based on Illumina short-reads with k = 21 kmer analysis. (**b**) The heatmap represents two pseudochromosomes of the *I. purchasi* genome.

In this study, we generated 61.21 Gb of Illumina short-read sequencing, 36.4 Gb of PacBio long-read sequencing, and 120.71 Gb of High-throughput chromosome conformation capture (Hi-C) sequencing. The final genome assembly size was 1,103.38 Mb, with a contig N50 of 12.81 Mb and scaffold N50 of 601.66 Mb (Fig. 1). The total length of the genome assembly was comparable to the estimated genome size (1,097.12 Mb) based on k = 21 kmer analysis (Fig. 2a). Following Hi-C assembly and manual adjustment of the heat map, a total sequence length of 1,102.86 Mb was mapped onto two chromosomes, representing 99.95% of the genome. Within the sequences mapped to chromosomes, the length of sequences with determined orientation and direction was 1,098.95 Mb, constituting 99.64% of the total length of the mapped chromosome sequences.

**Fig. 3:**
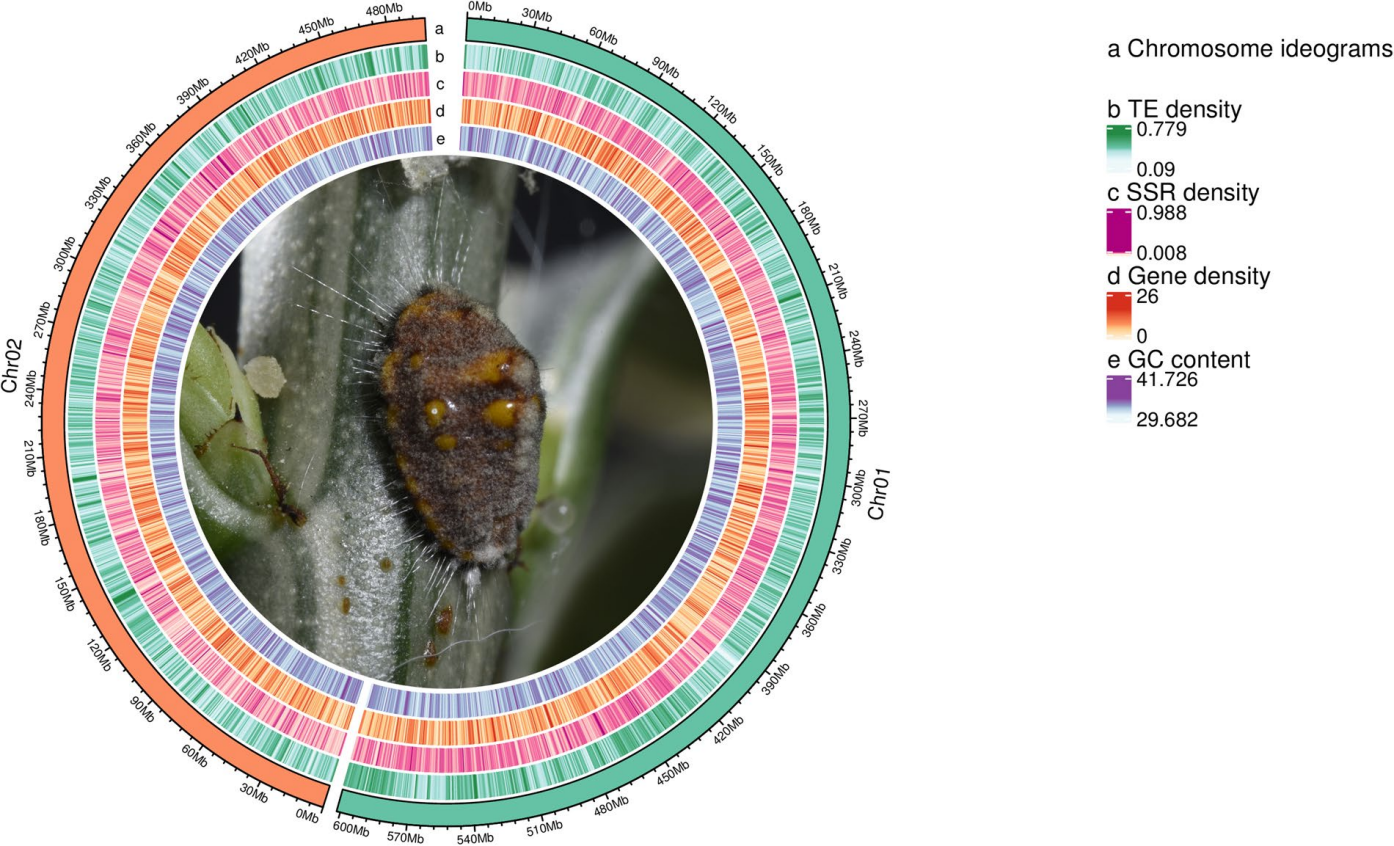
The circos plot describes the genomic characteristics of *I. purchasi*. Ecological photo is displayed in the center of the plot.

**Fig. 4:**
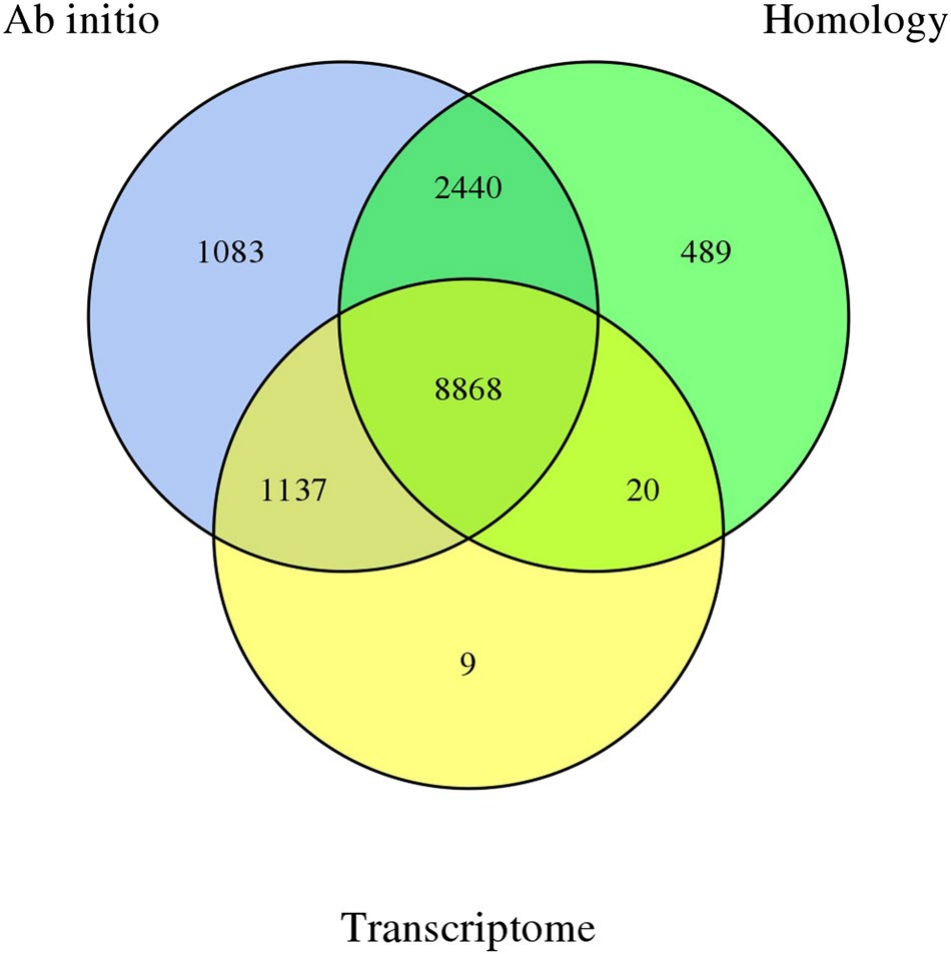
The Venn diagram showing the number of genes integrated by EVM that are supported by three prediction methods.

Based on the analysis of tandem repeats and interspersed repeats, we identified 462,722,633 bp of transposable element (TE) sequences, comprising 41.94% of the total, along with 35,341,500 bp of tandem repeat sequences, accounting for 3.20% (Fig. 3). Gene prediction involved homology-based prediction, ab initio prediction, and transcript-based prediction, resulting in a total of 14,046 predicted genes (Fig. 4). Further assessment through BUSCO analysis based on the insecta (odb_10) database indicated the presence of 93.20% in our predicted genes, indicating a high integrity of gene prediction. Annotation analysis of the predicted gene sequences against databases such as NR, EggNOG, GO, KEGG, SWISS-PROT, and Pfam yielded comprehensive annotation results, with a total of 89.71% of genes annotated to the databases (Table 1).

**Table 1:** Summary of genome annotations for *I. purchasi*.

Database	Number	Percentage (%)
GO	9,925	70.66
KEGG	10,316	73.44
KOG	8,342	59.39
Pfam	10,874	77.42
Swissprot	9,479	67.49
TrEMBL	12,401	88.29
eggNOG	10,125	72.08
NR	12,003	85.45
All	12,600	89.71

## Methods

### Sample collection and genome sequencing

*I. purchasi* was collected in 2020 from a lemon tree in Ningde, Fujian Province, China (26°34′47″N, 118°44′15″E). The laboratory colony was cultured on lemon trees for four generations in the insectarium under controlled conditions of 27 ± 1 °C and 60% relative humidity, with a photoperiod of 14:10 (L:D). Adult females and nymphs were collected for sequencing.

To perform genome sequencing, the samples were washed with 75% ethanol and sterile water, then stored in cryogenic vials with the addition of 95% ethanol. Subsequently, the total DNA from the entire body was extracted using the CATB method^9^ to construct three different sequencing libraries, followed by sequencing according to standard procedures. Firstly, for genome survey, we used the NEBNext® Ultra™ II DNA Library Prep Kit to build an Illumina short-read library, which was sequenced on the Illumina NovaSeq 6000 platform. Secondly, we constructed PacBio library using the SMRTbell Template Prep Kit and performed HiFi sequencing on the PacBio Sequel II platform. Finally, to generate a chromosome-level genome, we used the Mate-pair Kit to build Hi-C library, which was sequenced on the Illumina NovaSeq 6000 platform.

For transcriptome sequencing, we employed the Trizol method^10^ to extract RNA from fresh samples. The NEBNext® Ultra™ RNA Library Prep Kit was used to construct RNA library, which was sequenced on the Illumina NovaSeq 6000 platform for transcriptome sequencing. Additionally, the Ligation Sequencing Kit (SQK-LSK110; ONT) was used to construct library, and full-length transcriptome sequencing was completed on the Oxford Nanopore PromethION platform.

### Genome assembly

K-mer analysis was conducted using Jellyfish 2.1.4^11^ based on short-read data. Genomescope 2.0^12^ (-k 21 -p 2 -m 100,000) was used to estimate genome size and heterozygosity. The PacBio Sequel II platform generated a total of 36.4 Gb of long reads. These reads were assembled using Hifiasm v0.14^13^. For chromosomal scaffolding, Hi-C data were aligned to the contig-level genome sequence using Bwa v0.7.17^14^. Paired reads with mate mapped to a different contig were used to do the Hi-C associated scaffolding. Then, self-ligation, non-ligation and other invalid reads were filtered out. The uniquely mapped data were retained for assembly using LACHESIS^15^. Any two segments which showed inconsistent connection with information from the raw scaffold were checked manually. These corrected scaffolds were divided into subgroups and sorted and oriented into pseudomolecules with LACHESIS (CLUSTER_MIN_RE_SITES = 35; CLUSTER_MAX_LINK_DENSITY = 2; ORDER_MIN_N_RES_IN_TRUNK = 98; ORDER_MIN_N_RES_IN_SHREDS = 97). After this step, placement and orientation errors featuring obvious discrete chromatin interaction patterns were manually adjusted, and the Hi-C interaction heatmap was constructed using the ggplot2^16^ software in the R package. Finally, high quality assembly at chromosome level was obtained, and a snail plot of genomic features was generated by Blobotootlkit^17^.

### TE annotation

Transposon element (TE) was identified by a combination of homology-based and de novo approaches. Initially, de novo prediction was conducted using RepeatModeler2 v2.0.1^18^, which invoked RECON v1.0.8^19^ and RepeatScout v1.0.6^20^. The predicted results were categorized using RepeatClassifier^18^ with the assistance of the Dfam database v3.5^21^. Subsequently, LTR _retriever v2.9.0^22^ was utilized for de novo prediction specifically targeting Long Terminal Repeats (LTRs). Full-length long terminal repeat retrotransposons (fl-LTR-RTs) were identified using both LTRharvest v1.5.10^23^ and LTR_FINDER v1.07^24^ (ltr_finder -w 2 -C -D), resulting in the generation of high-quality intact fl-LTR-RTs and a non-redundant LTR library. The de novo prediction results were integrated with the known databases to construct a non-redundant species-specific TE library. Employing RepeatMasker v4.1.2^25^ (repeatmasker -nolow -no_is -norna -engine wublast -parallel 8 -qq), homology searches were performed on the library to identify and classify TE sequences in the final chromosome genomes. Concatenated repeat sequences were annotated by MIcroSAtellite identification tool (MISA v2.1)^26^ and Tandem Repeat Finder v4.09^27^ (trf 2 7 7 80 10 50 500 -d -h).

### Gene prediction and annotation

We integrated three approaches (ab initio prediction, homology search and transcript-based assembly) to annotate protein-coding genes in the genome. Ab initio prediction was conducted using Augustus v3.1.0^28^ and SNAP (2006-07-28)^29^. GeMoMa v1.7^30^ was utilized for Homology-based prediction, comparing gene features with those of cotton mealybug (*P. solenopsis*), the fruit fly (*Drosophila melanogaster*), pea aphids (*Acyrthosiphon pisum*), and tobacco whitefly (*Bemisia tabaci*). For transcript-based prediction, RNA sequencing data were mapped to the reference genome using Hisat v2.0. 4^31^ (hisat2–dta -p 10), and assembled using Stringtie v1.2.3^32^ (stringtie -p 2). GeneMarkS-T v5.1^33^ was employed for gene prediction based on assembled transcripts. The PASA v2.4.1^34^ was used to predict genes based on the unigenes (and full-length transcripts from the ONT sequencing) assembled by Trinity v2.11^35^ (Trinity–genome_guided_bam). Finally, predictions from these three methods were integrated using EVM v1.1.1^36^ and modified with PASA. Gene prediction completeness was assessed using BUSCO v5.2.2^37^ (busco -m prot). Gene functions were deduced through comprehensive sequence alignments against several databases including the NCBI Non-Redundant (NR), EggNOG, KOG, TrEMBL, InterPro, and Swiss-Prot, employing diamond blastp (diamond v0.9.29^38^) and the Kyoto Encyclopedia of Genes and Genomes (KEGG) database with an E-value threshold of 1E-3. Protein domains were annotated via InterProScan v5.34^39^ utilizing InterPro protein databases, while PFAM databases was used the identification of motifs and domains within gene models. Gene Ontology (GO) IDs for each gene were obtained from TrEMBL, InterPro, and EggNOG.

## Data Records

The raw data of genome and transcriptome sequencing were deposited in the Genome Sequence Archive^40^ (GSA, https://ngdc.cncb.ac.cn/gsa) at the National Genomics Data Center (NGDC) under the accession number CRA014119^41^. The genome sequence was stored in the Genome Warehouse^42^ (GWH, https://ngdc.cncb.ac.cn/gwh) with the accession number GWHERBG00000000. All data are linked to BioProject PRJCA022271. The genome sequence was also deposited in the National Center for Biotechnology Information (NCBI) GenBank, with accession number GCA_039619475.1^43^, under BioProject PRJNA1056491. The genome annotations are available from the Figshare repository^44^.

## Technical Validation

High mapping quality were confirmed in our results. After assembling PacBio reads, Bwa^12^ was used to align Illumina short-reads with the assembled genome, resulted in mapping rate of 99.36%. The coverage of the genome by Illumina short-reads is 99.86% with an average depth of 51. Minimap2^45^ was used to align HiFi reads with the assembled genome, resulted in mapping rate of 98.90%, and achieving a genome coverage of 99.98% with an average depth of 31. A genome preprint on *I. purchasi* demonstrated similar genome size and composition, with a genome size of 1,098.4 Mb and a GC content of 33.4%^46^.

## Data Availability

All software used in this study is in the public domain and its parameters are clearly described in the methodology. If the detailed parameters of the software are not mentioned, the default parameters are used as recommended by the developer. No custom code or scripts were used for the curation and validation of the dataset.
